# FUNDC1 predicts Poor Prognosis and promotes Progression and Chemoresistance in Endometrial Carcinoma

**DOI:** 10.7150/jca.96877

**Published:** 2024-10-21

**Authors:** Lihua Tang, Jiongyu Chen, Zhaoting Wu, Luanhong Wang, Yaozhen Lai, Zejia Chen, Lin Peng, Li Zhou

**Affiliations:** 1 Department of Gynecologic Oncology, Cancer Hospital of Shantou University Medical College, Shantou, China.; 2 Central Laboratory, Cancer Hospital of Shantou University Medical College, Shantou, China.; 3 Shantou Key Laboratory of Precision Diagnosis and Treatment in Women's Cancer, Shantou, China.; 4 Department of Gynecology, First People's Hospital of Chenzhou, Chenzhou, China.; 5 Department of Gynecology, Shenzhen Pingle Orthopedic Hospital (Shenzhen Pingshan Traditional Chinese Medicine Hospital), Shenzhen, China.

**Keywords:** FUN14 domain-containing protein 1, Endometrial cancer, Chemotherapy resistance, Prognostic biomarker, Mitophagy

## Abstract

Absence of effective prognostic biomarkers and therapeutic targets for reversing chemoresistance of endometrial carcinoma (EC) remains a huge challenge for clinicians. Mitophagy plays a crucial role in carcinogenesis and chemoresistance. FUN14 domain-containing protein 1 (FUNDC1) is a novel mitophagy receptor protein involved in tumorigenesis under hypoxic conditions. However, the implication of FUNDC1 in EC progression, chemoresistance in particular, remains unclear. Based on The Cancer Genome Atlas (TCGA) cohort, comprised of 403 EC patients, the association of FUNDC1 mRNA levels with hypoxia-inducible factor 1α (HIF-1α) expression, clinicopathologic features and prognosis in EC was analyzed, and subsequently verified utilizing immunohistochemistry of 288 EC specimens. Analysis of the cohort in TCGA showed that patients with higher FUNDC1 levels exhibited worse OS, with the shortest OS exhibited by patients with co-upregulated FUNDC1 and HIF-1α (*P* < 0.05). Analysis of the validation cohort indicated that OS and PFS rates of high-FUNDC1 patients were lower than that of low-FUNDC1 group (*P* < 0.05). Cases with co-downregulation of FUNDC1 and HIF-1α had higher OS and PFS rates than those with co-upregulation of these two proteins (88.8% vs. 71.2%, *P* = 0.002; 85.6% vs. 71.2%, *P* = 0.009). Higher FUNDC1 expression was observed in platinum-resistant patients. Multivariate Cox regression analysis revealed that FUNDC1 expression, FIGO stage, lymphatic invasion, depth of myometrial invasion, and ascites were independent risk factors for OS and PFS. Kyoto Encyclopedia of Genes and Genomes (KEGG) pathway enrichment analysis showed that FUNDC1 was closely related to spliceosome, neurodegeneration pathways of multiple diseases, and cell cycle signaling pathways. Significantly enriched RNA splicing and ncRNA processing were identified in Gene Ontology (GO) analysis. Gene set enrichment analysis (GSEA) indicated that abnormal expression of FUNDC1 was involved in endometrial cancer, NOD-like receptor signaling pathway and cytokine signaling in the immune system. In addition, immune cell infiltration analysis by Tumor Immune Estimation Resources (TIMER) database and the Xiantao academic tool demonstrated that FUNDC1 expression was strongly associated with the infiltration of Th2, NK, Th17, Tem, pDC, neutrophil, MDSC, CD4+ T, and γδ T cells. Knockdown of FUNDC1 using shRNA in HEC-1B and Ishikawa EC cells inhibited proliferation, migration and invasion, accompanied by enhanced chemotherapeutic susceptibility to carboplatin and paclitaxel. Accordingly, FUNDC1 could be a prospective prognostic biomarker and potential therapeutic target for EC.

## Introduction

Endometrial carcinoma (EC) is a prevailing malignancy affecting the female reproductive system, occurring with increasing incidence in younger individuals in recent years^1^. As per the GLOBCAN 2020 report, the new cases of endometrial carcinoma were estimated to be 417,000 globally, with an estimated 97,400 deaths in the year 2020^2^. Surgical resection is the primary therapeutic approach for EC, while chemotherapy is predominantly employed in patients with advanced disease or recurrent metastasis, as well as those presenting high-risk factors for recurrence following surgery. Nevertheless, the development of resistance to chemotherapy poses significant challenges^3^, resulting in therapeutic failure and mortality in over 90% of patients with advanced disease^4^, ^5^. The absence of effective indicators to predict EC prognosis and specific targets for reversing chemoresistance is a notable limitation.

Mitochondria are double-layered membrane organelles responsible for cellular energy production, functioning as the fulcrum of cellular homeostasis through regulating intracellular calcium, apoptosis, signal transduction, and redox balance^6^. Malfunctioning mitochondria usually induce high levels of reactive oxygen species (ROS), ultimately leading to deleterious oxidation of cellular DNA and proteins^7^-^11^. Mitophagy, a selective form of macroautophagy in which dysfunctional or fragmented mitochondria are efficiently degraded, is involved in tumorigenesis and progression of EC^12^, ^13^. Moreover, mitophagy has been demonstrated to be a protective mechanism underlying drug tolerance in cancer cells^14^. It has been reported that inhibition of BCL2 and adenovirus E1B 19-kDa-interacting protein 3 (BNIP3)-driven mitophagy counteracts cisplatin resistance in ovarian carcinoma and osteosarcoma^15^. However, only a few mitophagy-associated genes, such as *TOMM40*, and *KIF4A*, have been identified as genes that facilitate EC progression^12^, ^16^. Identification and development of additional prognostic biomarkers and efficacious targets for precise therapeutic interventions in EC is needed.

Mitophagy can be driven by two types of pathways: one is ubiquitin-mediated mitophagy, such as the PINK1/Parkin pathway, and the other is receptor-dependent, protein-mediated mitophagy^17^. Several selective receptors are involved in ligand-receptor recognition, including BNIP3, BNIP3-like (NIX or BNIP3L), prohibitin-2 (PHB2), and FUNDC1. Among them, FUNDC1 is a recently identified mitochondrial protein with conserved sequences from Drosophila melanogaster to Homo sapiens, and which induces receptor-mediated mitophagy by interacting with microtubule-associated protein light chain 3 (LC3) during hypoxia^18^, ^19^. Our previous research showed a correlation between high FUNDC1 expression and unfavorable prognosis in breast cancer. Moreover, a stimulative effect of FUNDC1 on cell proliferation was shown, which highlights its potential as a novel therapeutic target in breast cancer therapy^20^. FUNDC1 overexpression correlates with poor prognosis and therapeutic resistance in various human malignancies, such as cervical cancer, laryngeal cancer, hepatocellular carcinoma, pancreatic cancer, and urothelial cancer^21^-^25^. However, the implications of FUNDC1 in clinical outcomes of patients with EC, and the roles and mechanisms of FUNDC1 in EC progression and chemoresistance remain unclear. Notably, HIF-1α/FUNDC1 mitophagy has been indicated to be dependent on activation of a hypoxia inducible factor-1α (HIF-1α)/BNIP3/FUNDC1 signaling pathway^26^. Herein, we assess the correlation between expression of FUNDC1 and HIF-1α, and its correlation with survival time and clinicopathological parameters among patients with EC, and explore the possible mechanisms of FUNDC1 in the susceptibility of EC cells to carboplatin and paclitaxel treatment *in vitro*.

## Materials and Methods

### Data download and tissue specimen collection

RNA-seq transcriptome profiling and corresponding clinical information for 403 endometrial adenocarcinoma samples were obtained from The Cancer Genome Atlas (TCGA) (https://portal.gdc.cancer.gov/). Tumor tissue was collected from 288 EC patients who underwent surgical resection between January 2002 and September 2012 and were confirmed by pathological diagnosis, and served as the validation set from the Cancer Hospital of Shantou University Medical College. Among them, 92 patients received platinum-based chemotherapy after surgery. The cases were included based on the following criteria: 1) no previous malignant disease or a second primary tumor, and 2) no prior chemotherapy or radiotherapy history. Clinical parameters, including age, body mass index (BMI), Federation of International of Gynecology and Obstetrics (FIGO) stage, tumor grade, invasion status, menstrual status, disease history, CA125, Lactate dehydrogenase (LDH), expression of Estrogen receptor (ER) and Progesterone receptor (PR), and follow-up were obtained from medical records. The research protocol was approved by the Institute Research Ethics Committee of the Cancer Hospital of Shantou University Medical College.

### Cell culture and treatment

EC cell lines HEC-1B and Ishikawa were purchased from the Fuheng Cell Center (Shanghai, China). Cells were maintained in complete RPMI 1640 medium (Gibco, CA, USA) supplemented with 10% fetal bovine serum (FBS, Biological Industry, Kibbutz Beit HaEmek, Israel), and cultured at 37℃ in a humidified 5% CO_2_ incubator. Short hairpin RNA (shRNA) was used to silence the expression of FUNDC1 by infecting with recombinant lentivirus particles encoding shRNA targeting FUNDC1 (shFUNDC1#1: 5'-GAAAGTGATGACGACTCTTAT-3'; shFUNDC1#2: 5'-GATTAAGAAACGAGCGAACAA-3', Beijing SyngenTech Co., LTD). Control cells were transduced with control lentiviral shRNA (shNC:5'-TTCTCCGAACGTGTCACGTTT-3', Beijing SyngenTech Co., LTD). To investigate the effect of FUNDC1 in cellular response to chemotherapy drugs, cell viability was evaluated by CCK8 assay after treatment with carboplatin (0, 1.25, 2.5, 5, 10, 20, 50 μg/ml; Qilu Pharmaceutical Co.) and paclitaxel (0, 0.5, 1, 2.5, 5, 10, 20 μg/ml; Huiyu Pharmaceutical Co.) for 24, 48 and 72 hours.

### Immunohistochemistry and evaluation

We performed immunohistochemistry (IHC) to determine FUNDC1 and HIF-1α expression in EC tissue specimens. Primary antibodies against FUNDC1 (1:500 dilution, Bioss, Beijing, China), and HIF-1α (1:200 dilution, Abcam, Cambridge, UK) were used along with secondary antibodies obtained from Fuzhou Maixin Biotechnology Ltd. Three experienced pathologists independently evaluated the IHC results by a semi-quantitative scoring method, with the values for staining intensity (negative, 0; weakly positive, 1; moderately positive, 2; and intensely positive, 3) and the proportion of positive cells (<25%, 1; 25-50%, 2; 51-75%, 3; and 76-100%, 4). The staining index was determined by multiplying both score sets, and ranged from 0-12. The optimal cut-off was determined using X-tile software (3.6.1, Yale), after which patients were divided into low-expression and high-expression groups.

### Cell viability assay

Cell viability was assessed using a Cell Counting Kit-8 (CCK8) according to the manufacturer's instructions. In brief, EC cells were cultured in 96-well plates at a density of 5000 cells per well overnight at 37°C in a 5% CO_2_ incubator. The next day, the cells were exposed to varying concentrations of paclitaxel or cisplatin for 24, 48, or 72 h. Subsequently, 10 µl of CCK-8 solution was added to each well, and cells were incubated for 3 h. Light absorbance of the plates was measured at 450 nm using a microplate reader (MK3, Thermo, USA). All experiments were performed in triplicate.

### Wound healing assay

A wound healing assay was utilized to assess cell migratory capacity. Cells were seeded in a 6-well plate and then cultured until confluence was reached. Afterward, a scratch was created using a 20 μl pipette tip, and the cells were incubated in RPMI 1640 medium for 24 h. Multiple photographs were captured at pre-marked locations, both at 0 and 24 h. The lesion area was quantified using an Image-J analysis system. Experiments were replicated three times.

### Invasion assay

Cell invasion was determined using 8 µm pore diameter, Matrigel-coated transwell invasion chambers (BIOFIL, Guangzhou, China). The lower chamber was filled with 600 µl of RPML 1640 medium containing 10% FBS as a chemoattractant. A total of 1 × 10^5^ cells from each group were seeded in the upper chamber (in 200 µl of serum-free RPMI 1640 with 1% BSA). After a duration of either 18 or 24 h, the upper chamber and the cells situated on the upper side of the membrane were removed. Cells adhering to the lower surface were stained with 0.1% crystal violet and counted, in five predetermined fields, (40×) under a microscope (Model: DM3000, Leica, Germany). The assay was repeated three times.

### Western blotting

Protein extraction was performed using lysis buffer (including phosphatase inhibitors and PMSF). A BCA Protein Assay kit (Solarbio, Beijing, China) was used to determine the total protein concentration. Total protein (30 µg) was loaded onto an SDS-PAGE gel, and then transferred to a PVDF membrane after electrophoresis. Subsequently, PVDF membranes were blocked with 5% BSA (Solarbio, Beijing, China) in TBST for 2 h at room temperature, then incubated with primary antibodies at 4℃ overnight. Antibodies used for western blotting included the following: rabbit anti-FUNDC1 antibody, rabbit anti-LC3B antibody, mouse anti-β-Actin antibody (all 1:1000 dilution, Cell Signaling Technology, MA, USA), and rabbit anti-HIF-1α antibody (1:1000 dilution, Abcam, Cambridge, UK). Secondary antibodies were HRP-conjugated goat anti-rabbit antibody and anti-mouse antibody (1:1000 dilution, Cell Signaling Technology, MA, USA). Each experiment was performed in triplicate.

### Mitophagy characterization

Cells were grown on slides and cultured at 37℃ for 24 h. After treatment and washing with PBST, cells were prestained with 200 nM MitoTracker Red CMXRos (Beyotime, Nanjing, China) for 30 min at 37℃, to label the mitochondria, followed by 75 nM LysoTracker Green (Beyotime, Nanjing, China) for 30 min, as previously described^27^, ^28^. Then the MitoTracker Red CMXRos and LysoTracker Green working solution was removed and DAPI was added for staining nuclei. Then, the cells were washed with PBST and sealed with cover glasses. Subsequently, fluorescent images were observed under the fluorescence microscope (Model: BX51, OLYMPUS, Japan). The co-localization of mitochondria and lysosomes was assessed by Pearson's correlation coefficient analysis using ImageJ software. For detection of mitochondrial membrane potential, cells were cultured with JC-1 for 30 min and fluorescence intensities of JC-1 aggregates (red) and JC-1 monomers (green) were ultimately measured using a fluorescence microscope (OLYMPUS BX51, Japan). The ratio of JC-1 aggregates to monomers represents the mitochondrial membrane potential. Fluorescence images were analyzed by Image J and GraphPad Prism 8.

### Enrichment analysis of FUNDC1-associated genes

The Xiantao academic online analysis tool (https://www.xiantaozi.com/) was used to identify the first 930 genes (with a Spearman's correlation coefficient at an absolute value above 0.5) associated with FUNDC1 in the EC TCGA database. Gene Ontology (GO) and Kyoto Encyclopedia of Genes and Genomes (KEGG) analyses were employed to annotate the functions of these genes related to FUNDC1. Gene set enrichment analysis (GSEA) was performed utilizing the Xiantao academic database in order to further investigate the molecular mechanism and signaling pathway of FUNDC1. The |normalized enrichment score (NES)| > 1, adjusted* P* < 0.05 and false discovery rate (FDR) < 0.25 was considered to indicate significant enrichment.

### Immune infiltration analysis

The association between FUNDC1 expression and immune cell infiltration in EC was explored by using the Tumor Immune Estimation Resources (TIMER) database (Version 2.0 http://timer.comp-genomics.org/timer/) and the Xiantao academic online analysis tool.

### Statistical analysis

All statistical analyses were performed using SPSS 24.0 statistical software, and images were processed using GraphPad Prism8 and R 4.1.2. The optimal cutoff value for continuous variables was determined using X-tile software (3.6.1, Yale). The chi-square test or the Student's t-test was adopted for comparing differences among groups. Pearson's correlation analysis was employed to assess the correlation between HIF-1α and FUNDC1 expression. Kaplan-Meier analysis and log-rank tests were performed for survival analysis. Univariate and multivariate Cox proportional hazards regression models were used to identify independent prognostic factors. Cox multivariate regression analysis (forward method) was used to assess all parameters in the univariate analysis. All tests were two-sided, and a *P* < 0.05 was considered significant.

## Results

### Correlation of FUNDC1 and HIF-1α expression in EC

**Figure 1:**
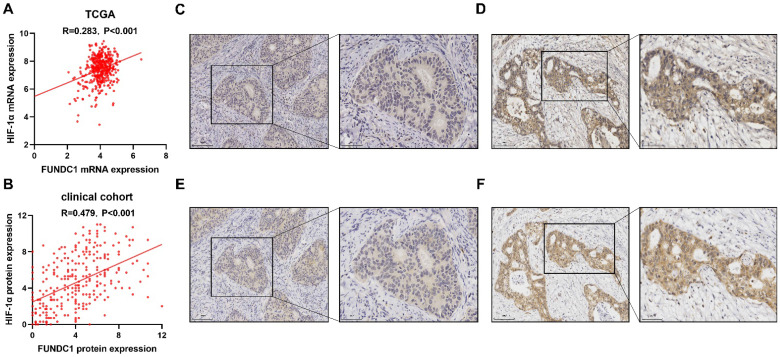
Expression of FUNDC1 and HIF-1α in EC. Relationship of FUNDC1 and HIF-1α in the EC TCGA cohort (**A**) and clinical cohort (**B**). Serial sections of 288 endometrial carcinoma tissues from our cohort were stained with FUNDC1 and HIF-1α antibodies. Representative image of low (**C**) and high (**D**) FUNDC1 expression. Representative image of low (**E**) and high (**F**) HIF-1α expression.

A total of 403 patients were obtained from TCGA and included and categorized into sub-groups of high and low mRNA expression for FUNDC1 and HIF-1α. Co-upregulation of HIF-1α and FUNDC1 were observed in 144 (35.7%) samples, whereas 56 (13.9%) cases displayed co-downregulation. Pearson correlation analysis showed that FUNDC1 was positively associated with HIF-1α (*R* = 0.283, *P* < 0.001;** Figure 1A**). Then, we evaluated the protein expression of FUNDC1 and HIF-1α in our cohort of 288 EC patients by IHC staining. FUNDC1 was predominantly located in the cytoplasm of tumor cells (**Figure 1C; Figure 1D**), and HIF-1α was detected in the cytoplasm and nucleus (**Figure 1E; Figure 1F**). Among these cases, 125 (43.4%) specimens exhibited co-downregulated levels of FUNDC1 and HIF-1α, and 66 (22.9%) with co-upregulation. In addition, the expression of FUNDC1 was positively correlated with HIF-1α (*R* = 0.479, *P* < 0.001; **Figure 1B**).

### Association between FUNDC1 expression and clinicopathologic features

No significant difference in FUNDC1 mRNA expression was found among varying FIGO stages, tumor grades, or pelvic lymph node metastasis in the EC TCGA cohort (**Table S1**, all *P* > 0.05), except for age (χ^2^ = 5.38, *P* = 0.02). Immunohistochemically, no significant difference in protein expression of FUNDC1 was observed in any of the various clinicopathologic features in our cohort of 288 EC patients (**Table 1**).

### Correlation of FUNDC1 and HIF-1α with prognosis of EC

**Figure 2:**
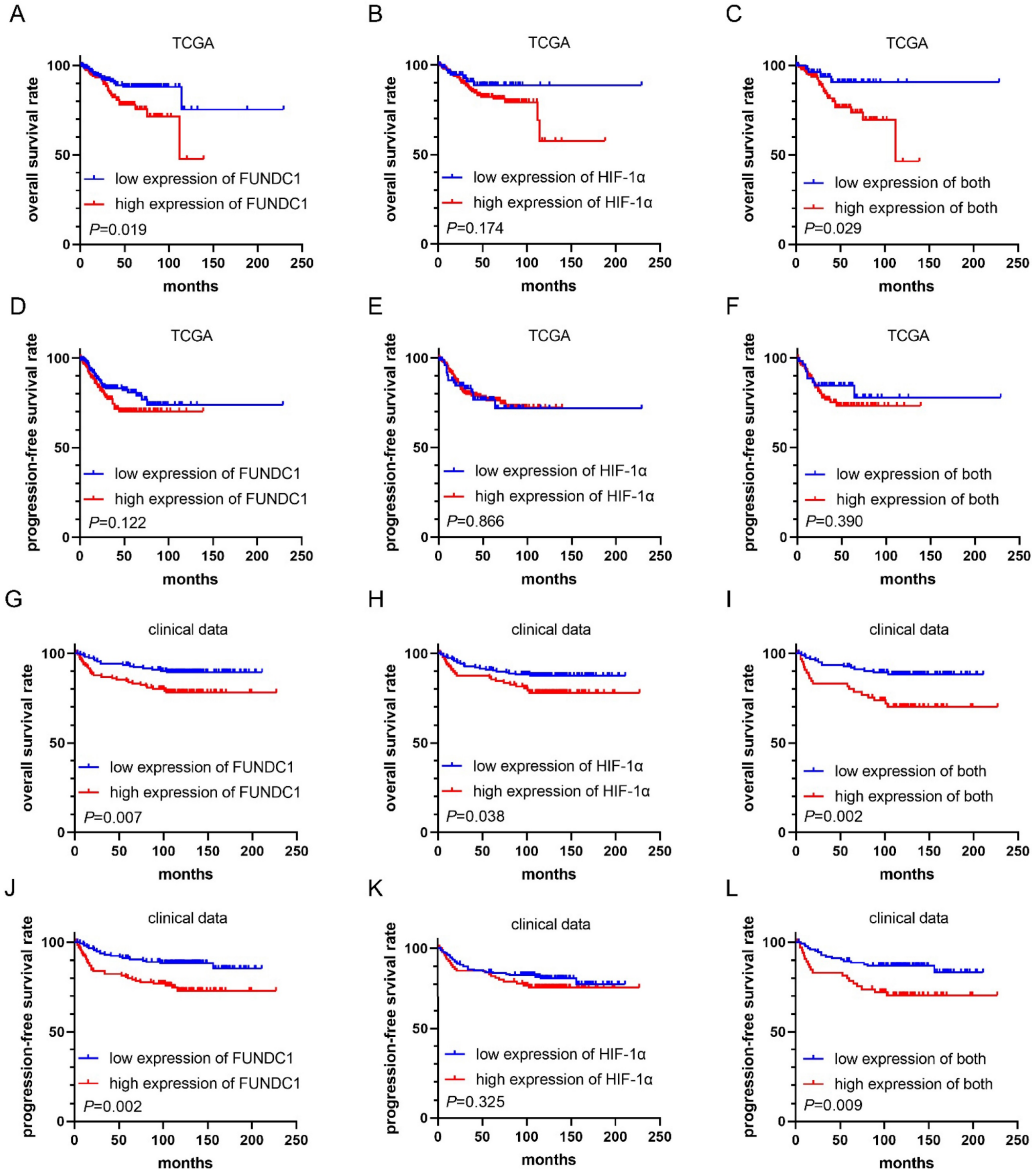
Kaplan-Meier curves for overall survival (OS) and progression-free survival (PFS) in EC patients. Comparison of OS **(A)** and PFS **(D)** between high FUNDC1 and low FUNDC1 groups in TCGA. Comparison of OS **(B)** and PFS **(E)** between high and low HIF-1α groups in TCGA. Comparison of OS **(C)** and PFS **(F)** between high and low HIF-1α and FUNDC1 co-expression groups in TCGA. Comparison of OS **(G)** and PFS **(J)** between high FUNDC1 and low FUNDC1 groups in clinical data. Comparison of OS **(H)** and PFS **(K)** between high and low HIF-1α groups in clinical data. Comparison of OS **(I)** and PFS **(L)** between high and low HIF-1α and FUNDC1 co-expression groups in clinical data.

Analysis of the EC TCGA cohort showed that patients with higher FUNDC1 levels in EC tissues exhibited worse OS (*P* = 0.019, **Figure 2A**), while no significant association was found between HIF-1α and OS (**Figure 2B**). Notably, the subgroup with co-upregulation of FUNDC1 and HIF-1α showed shorter OS than those with co-downregulation (*P* = 0.029, **Figure 2C**). However, no significant correlation was found between PFS and FUNDC1, HIF-1α nor their co-expression (**Figure 2D-F**).

In our cohort, the median duration of follow-up for all patients was 115 months, and ranged from 2 to 227 months. For patients with high FUNDC1 expression, the OS and PFS rates were 78.6% and 74.8%, respectively, compared to those with low FUNDC1 expression (89.8%, *P* = 0.007; 87.3%, *P* = 0.002; **Figure 2G and J**). High HIF-1α expression was correlated with unfavorable OS of EC patients (*P* = 0.038, **Figure 2H**). Moreover, cases with co-downregulation of FUNDC1 and HIF-1α had higher OS and PFS rates than those with co-upregulation of these two proteins (88.8% vs. 71.2%, *P* = 0.002; 85.6% vs. 71.2%, *P* = 0.009; **Figure 2I and L**). Univariate Cox proportional hazard regression analysis indicated that FUNDC1 expression was negatively associated with OS and PFS, and HIF-1α was inversely correlated with OS (**Table 2**). Multivariate Cox regression analysis revealed that FUNDC1 expression, FIGO stage, lymphatic invasion, depth of myometrial invasion, ascites were independent risk factors for OS and PFS of EC patients, as shown in **Table 2 and 3** (HR 2.46, 95% CI: 1.13-5.35, *P*=0.023; HR 2.67, 95% CI: 1.32-5.39, *P*=0.006).

### Silencing FUNDC1 inhibits the proliferative and metastatic phenotypes of EC cells

**Figure 3:**
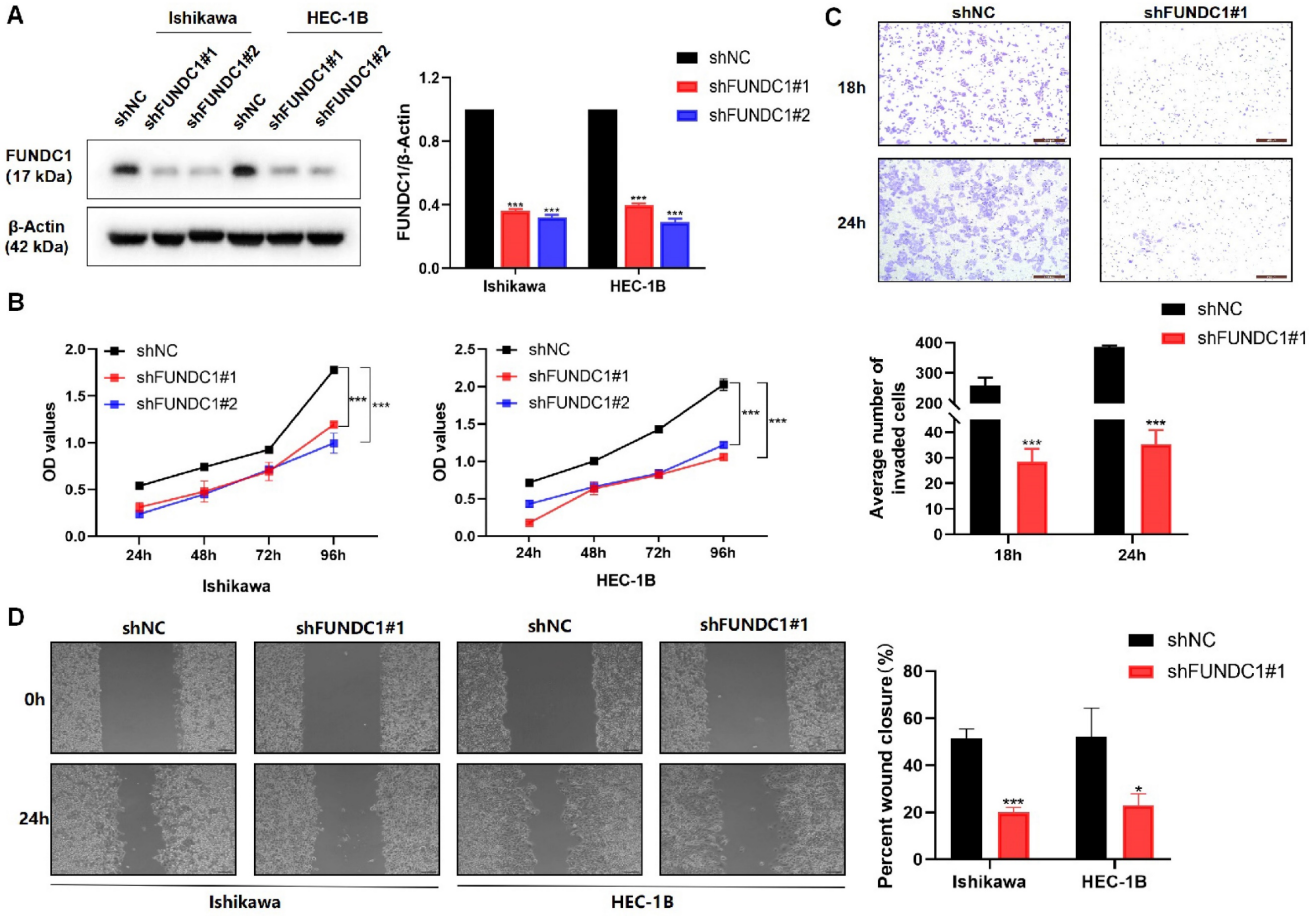
Silencing of FUNDC1 inhibits the viability and metastasis of EC cells. **(A)** For stable FUNDC1 knockdown, EC cells were transduced separately with two shRNAs (shFUNDC1#1 and shFUNDC1#2) that target FUNDC1 mRNA. Western blotting was used to detect the expression of FUNDC1 levels. **(B)** Cell growth rate was suppressed by FUNDC1 knockdown in Ishikawa and HEC-1B cells, as measured by CCK8 assay. **(C)** Cell invasion of HEC-1B and HEC-1B-shFUNDC1#1 cells. Representative images of cell invasion are shown on the right side, scale bar: 200 μm. **(D)** Wound healing assays were performed to characterize changes in the migration of HEC-1B, HEC-1B-shFUNDC1#1, Ishikawa and Ishikawa-shFUNDC1#1 cells. Scale bar: 100 μm. Results are expressed as mean ± SD of three independent experiments. *P < 0.05; **P < 0.01; ***P < 0.001.

Encouraged by the above data suggesting FUNDC1 is likely to contribute to EC progression, we next silenced the expression of FUNDC1 with shRNA (**Figure 3A**) and then assessed cell proliferation, invasion, and migration. Knockdown of FUNDC1 dramatically reduced the cell viability of EC cells (*P* < 0.01, **Figure 3B**). Compared to the controls, a decrease in the number of invading and migrating cells was observed (*P* < 0.05, **Figure 3C and D**).

### FUNDC1 knockdown increases susceptibility to carboplatin and paclitaxel in EC cells

**Figure 4:**
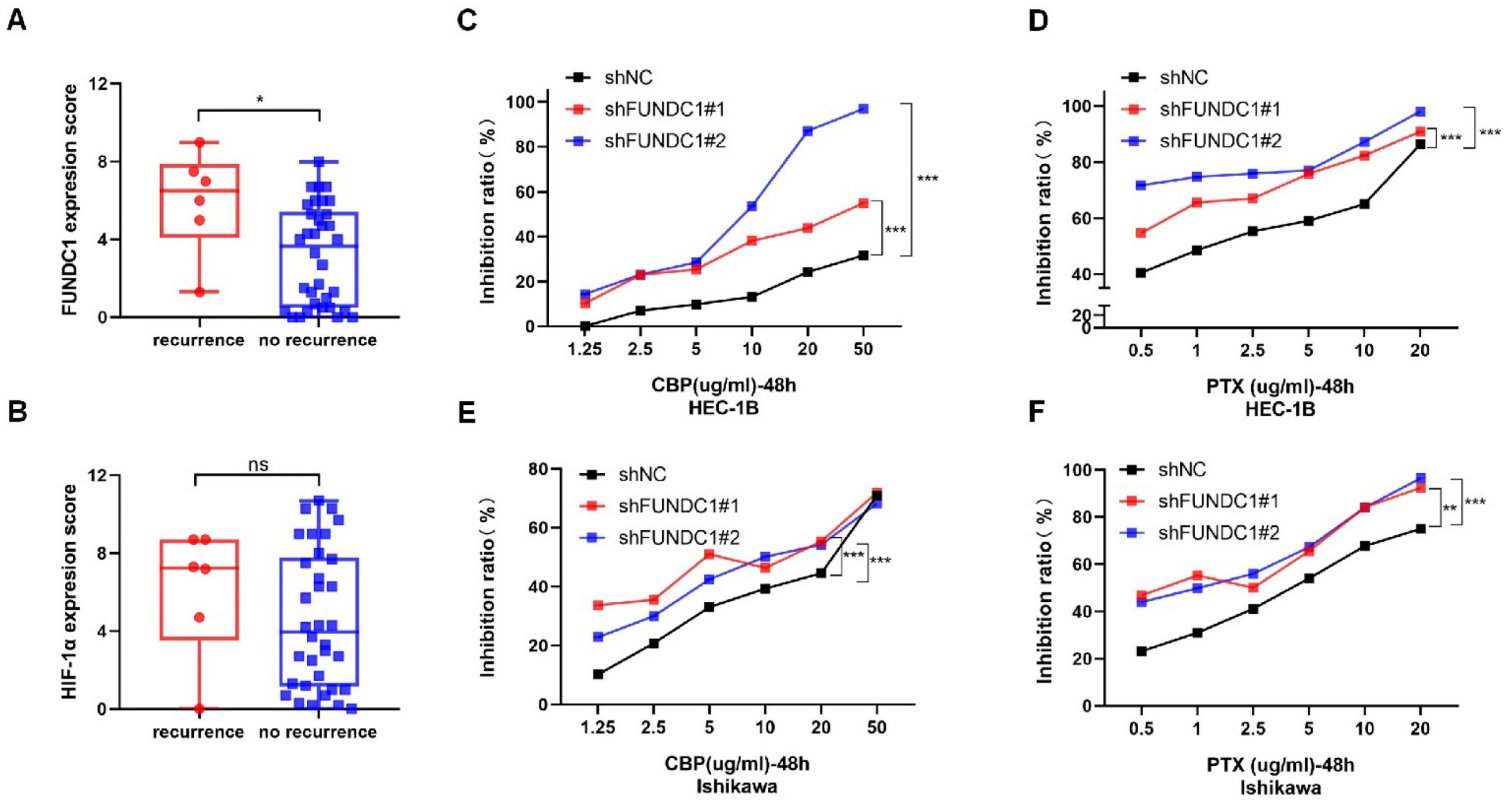
Relationship between FUNDC1 expression and chemoresistance. A comparison of FUNDC1 **(A)** and HIF-1α **(B)** IHC scores of EC patients with recurrence within 1 year after chemotherapy and those without recurrence. CCK8 assays were performed before and after retreatment with different concentrations of CBP for 48 h in HEC-1B cells **(C)** and Ishikawa cells **(E)**, as well as their FUNDC1-silenced cells. Different concentrations of PTX were used to treat HEC-1B, HEC-1B-shFUNDC1#1/2 cells **(D)**, Ishikawa and Ishikawa-shFUNDC1#1/2 cells **(F)**. Then, after 48 h, growth inhibition was assessed. *P < 0.05; **P < 0.01; ***P < 0.001.

To explore the role of FUNDC1 in chemoresistance of EC *in vitro*, we compared the cellular response to chemotherapeutic agents between HEC-1B-shFUNDC1 cells and controls. HEC-1B-shFUNDC1#1/2 cells consistently exhibited greater sensitivity to carboplatin- and paclitaxel-mediated toxicity at various concentrations (*P* < 0.01, **Figure 4C and D**). The same phenomenon was observed between Ishikawa and Ishikawa-shFUNDC1#1/2 cells (*P* < 0.01, **Figure 4E and F**). These results suggest that FUNDC1 inhibition can rescue chemosensitivity to carboplatin and paclitaxel chemotherapeutic agents in EC cells.

### High FUNDC1 levels correlate with platinum-based chemotherapy resistance in EC patients

Ninety-two EC patients in our cohort had received platinum-based chemotherapy after surgery. Among them, 40 patients underwent postoperative adjuvant chemotherapy alone and 52 patients received concurrent radiotherapy. Patients receiving only adjuvant chemotherapy were enrolled. We categorized those who experienced a recurrence within one year of chemotherapy as the “recurrence group” (chemo-resistance group), and those without relapse within one year were classified as the “no recurrence group” (chemo-sensitive group). As a result, a significant elevation in FUNDC1 levels was observed in the recurrence group compared to the sensitive group (*P* = 0.020, **Figure 4A**), suggesting that high FUNDC1 expression correlated with platinum-based chemotherapeutic resistance in EC patients. In contrast, HIF-1α expression was not significantly associated with chemotherapeutic tolerance (*P* = 0.328, **Figure 4B**).

### Depletion of FUNDC1 alleviates mitophagy in EC cells

**Figure 5:**
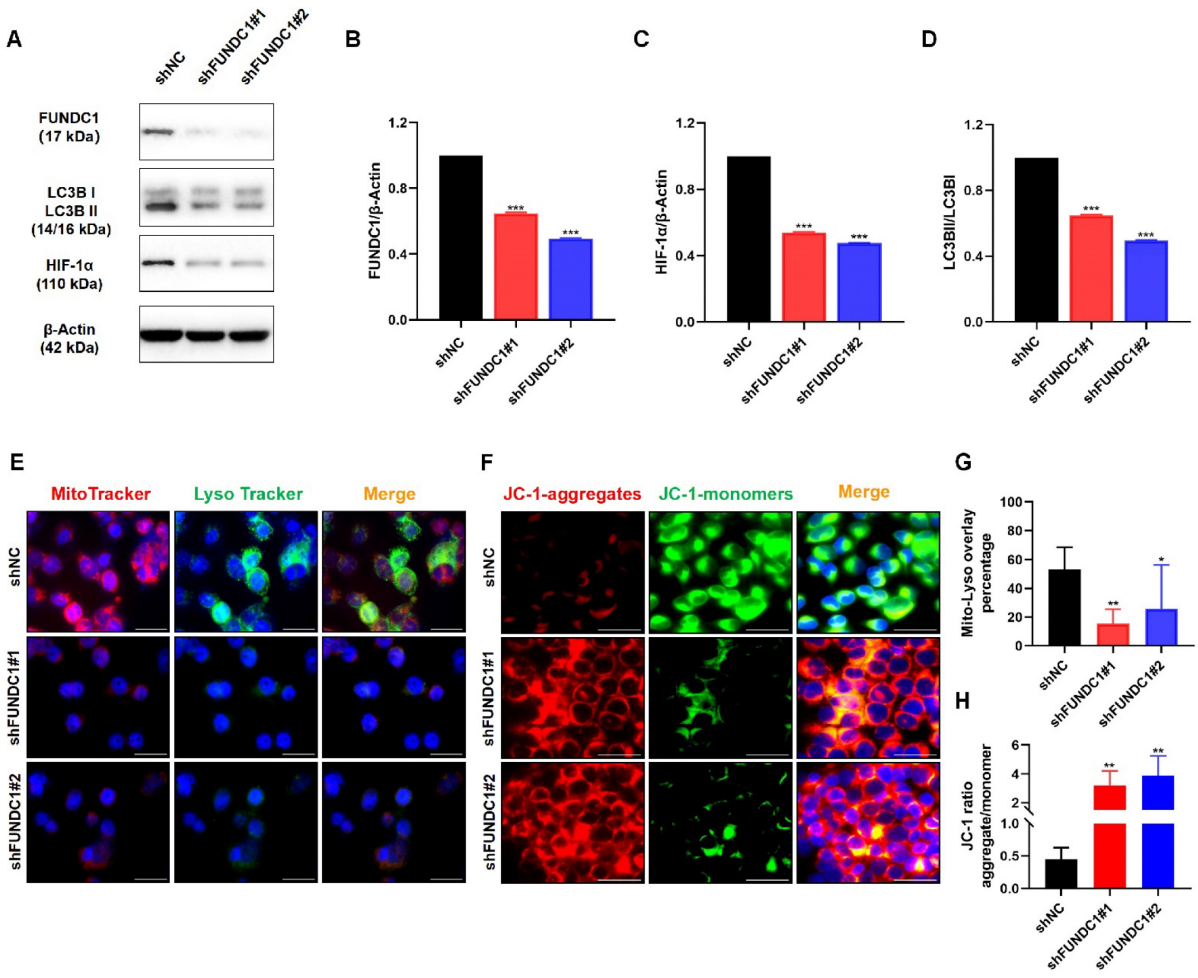
Inhibition of FUNDC1 impairs the expression of LC3B and HIF-1α, and decreases mitophagy in EC cells. **(A)** Western blot analysis was conducted to determine the protein levels of FUNDC1, HIF-1α, and LC3B, and the ratio of LC3BII to LC3BI. Quantitative analysis of western blots for FUNDC1 **(B)**, HIF-1α **(C)**, and LC3BII/LC3BI **(D)**. **(E)** HEC-1B and HEC-1B-shFUNDC1#1/2 cells were stained with both MitoTracker Red and LysoTracker Green, and were observed by fluorescence microscopy. The number of yellow puncta (mitochondria-lysosome colocalization) represents the formation of autolysosomes with internalized mitochondria. **(F)** The mitochondrial membrane potential of HEC-1B and FUNDC1-silenced HEC-1B cells is represented by JC-1 staining. Representative fluorescence images of JC-1 aggregates (red) and JC-1 monomers (green) are shown in the figure. **(G)** Quantification of the percentage of mitochondria-lysosome colocalization for HEC-1B and HEC-1B-shFUNDC1#1/2 cells. **(H)** Quantification of mitochondrial membrane potential (JC-1 aggregate/monomer ratio) for HEC-1B and HEC-1B-shFUNDC1#1/2 cells. Scale bar: 50 μm. n=3 per group. Data represent the mean ± SD. *P < 0.05; **P < 0.01; ***P < 0.001.

Since mitophagy contributes to chemoresistance and FUNDC1 is a key protein involved in mitophagy, we further evaluated the effects of FUNDC1 knockdown on mitochondrial function in EC cells. As expected, silencing FUNDC1 inhibited the expression of HIF-1α (**Figure 5A and C**), a crucial target gene involved in the mitophagy signaling pathway^29^, ^30^. FUNDC1 knockdown also reduced the conversion of LC3Ⅰ to LC3BⅡ (**Figure 5A and D**). Silencing FUNDC1 decreased the colocalization of mitochondria and lysosomes (*P* < 0.05, **Figure 5E and G**). Moreover, a higher JC-1 red/green ratio was observed in the HEC-1B-shFUNDC1 cells than in the controls (*P* < 0.05, **Figure 5F and H**). These results suggest that the inhibition of FUNDC1 could cause a decline in mitophagy in EC cells.

### Functional enrichment analysis of FUNDC1-related genes

**Figure 6:**
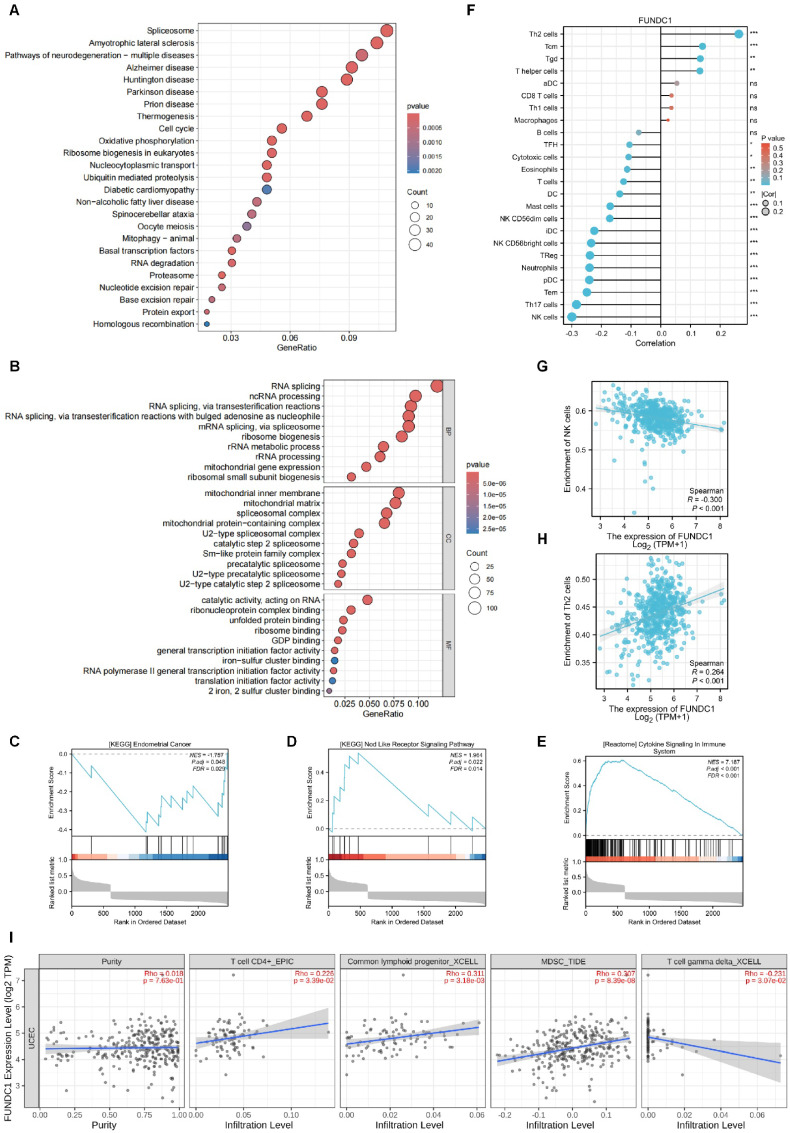
Functional enrichment analysis of FUNDC1-related genes and the relationship between FUNDC1 mRNA expression and immune infiltration in EC. KEGG (**A**) and GO (**B**) analyses for the top 930 FUNDC1-related genes in EC. The roles of FUNDC1 in EC based on GSEA (**C-E**). Expression of FUNDC1 was involved in endometrial cancer (**C**), NOD-like receptor signaling pathway (**D**) and immune system cytokine signaling (**E**). (**F**) Correlation between 24 immune cell abundances and FUNDC1 expression levels, with dot size indicating Spearman R value. Diagrams show correlation between NK cells (**G**), Th2 cells (**H**) infiltration levels and FUNDC1 expression. (**I**) Correlation analysis of FUNDC1 expression and immune infiltration in EC. *P < 0.05; **P < 0.01; ***P < 0.001.

Utilizing the Xiantao academic online analysis tool, the genes interacting with FUNDC1 were identified and the top 930 related genes were chosen for further functional enrichment analysis. KEGG analysis showed that the core FUNDC1-related genes mainly correlated with spliceosome, neurodegeneration of multiple diseases, and cell cycle signaling pathways (**Figure 6A**). For GO analysis, core genes were mostly involved in RNA splicing and ncRNA processing (**Figure 6B**). Gene set enrichment analysis (GSEA) analysis indicated that abnormal expression of FUNDC1 was involved in endometrial cancer, NOD-like receptor signaling pathway and cytokine signaling in the immune system (**Figure 6C-E**).

### The connection between FUNDC1 expression and immune cell infiltration

To investigate the potential immunomodulatory role of FUNDC1 in the tumor microenvironment, we explored the association between FUNDC1 expression and the tumor-infiltrating immune cells in TCGA cohorts. Xiantao revealed Th2 cells, central memory T cells (Tcm) cells, and Tgd cells were significantly increased in the group with high expression of FUNDC1. Conversely, natural killer (NK) cells, Th17 cells, effector memory T (Tem) cells, pre-dendritic (pDC) cells, neutrophils, and Treg cells showed negative relationships with FUNDC1 expression (**Figure 6F**, *P* < 0.05). Among them, NK cells and Th2 cells exhibited the most significant association, with correlation coefficients of -0.3 and 0.264, respectively (all *P* < 0.001, **Figure 6G-H**). In addition, TIMER analysis uncovered that common lymphoid progenitor, myeloid-derived suppressor cells (MDSCs) and CD4+ T cells were positively correlated with FUNDC1 expression but noted a negative association with γδ T cells (**Figure 6I**).

## Discussion

We investigated the potential role of FUNDC1 in EC progression and chemoresistance, based on tumor specimen analyses and *in vitro* experiments. This is the first study to demonstrate that the expression of mRNA and protein of FUNDC1 is elevated in EC tissues and is correlated with an unfavorable prognosis. *In vitro*, knockdown of FUNDC1 in EC cells resulted in a substantial reduction in proliferation, migration, and invasion, along with increased susceptibility to chemotherapeutic agents. These findings suggest a predictive value of FUNDC1 for EC prognosis, and the potential of targeting FUNDC1 as a therapeutic strategy for EC treatment.

High expression of FUNDC1 is an independent prognostic factor for shorter OS and PFS in patients with cervical cancer^23^. Moreover, depletion of FUNDC1 in cervical cancer cells inhibited cell viability and enhanced sensitivity to cisplatin and ionizing radiation^23^. A previous study in hepatocellular carcinoma also indicated the involvement of FUNDC1 in tumor progression and tumor immune microenvironment regulation^21^. Likewise, our previous study reported that high expression of FUNDC1 in breast cancer tissues is associated with poor outcomes and positively correlates with tumor size, stage and metastasis^20^. In addition, FUNDC1 could stimulate cell proliferation, migration and invasion *in vitro*^20^. Based on the above results, the present study demonstrates that patients with high FUNDC1 mRNA and protein expression exhibit worse OS. It is worth noting that EC patients with high FUNDC1 expression tended to show a higher incidence of pelvic lymph node metastasis than those with low FUNDC1 expression (14.50% vs 9.55%), suggesting a potential role of FUNDC1 in facilitating the spread of EC. Nevertheless, our findings suggest that FUNDC1 could be considered as a prognostic predictor and a therapeutic target for chemoresistance in EC.

Hypoxia is a prevalent feature in most solid tumors and is associated with aggressive phenotypes and therapeutic resistance^31^. A meta-analysis comprising 25 studies demonstrated a significant elevation in HIF-1α protein expression in EC tissues compared to that in normal tissues. Moreover, high HIF-1α expression predicts poorer prognosis and is associated with tumor grade, lymph node metastasis, and myometrial infiltration in EC patients^32^. Abundant evidence shows that mitophagy is closely associated with ROS and HIF-1α, which are produced by hypoxic stress^33^, ^34^. A recent study in hypoxic pulmonary hypertension indicated that FUNDC1-mediated mitophagy followed by ROS-HIF1α pathway activation contributed to the proliferation of pulmonary artery smooth muscle cells^35^. In agreement with the previous reports, our current investigation also established a positive correlation between FUNDC1 and HIF-1α in EC both in TCGA and our cohort, and demonstrated a correlation between co-expression of FUNDC1 and HIF-1α with OS and PFS in our cohort. Moreover, knockdown of FUNDC1 induced decreased expression of HIF-1α protein in EC cells, which is consistent with the aforementioned finding that FUNDC1-mediated mitophagy could activate the ROS-HIF1α pathway^35^. Therefore, we infer that the interaction of HIF-1α and FUNDC1 contributes to the carcinogenic mechanism of EC. It is worth noting that the upregulation of FUNDC1 was indicated to be blocked by HIF-1α inhibitor treatment in renal tubular cells, implying FUNDC1 also serves as a downstream regulator of HIF-1α^36^. Hence, the involvement of HIF-1α/FUNDC1 signaling in EC progression is still a disputed issue and needs to be clarified further.

FUNDC1 was identified as a mitochondrial outer membrane protein implicated in mitophagy by interacting with LC3B via its LC3 interaction region^37^, ^38^. In the case of hypoxia, phosphorylation of FUNDC1 at Ser17 facilitates mitophagy by boosting the interaction between FUNDC1 and LC3, resulting in a pivotal link between autophagosomes and fragmented mitochondria^39^. Conversely, dephosphorylation of FUNDC1 at Ser13 and at Tyr18 also induces the interaction of FUNDC1 with LC3, ultimately leading to the initiation of mitophagy^39^, ^40^. LC3B exists as type I and type Ⅱ, with the level of LC3BⅡ generally being related to autophagy activity. In the current study, we found FUNDC1 depletion induced reduction in the LC3BⅡ/Ⅰ ratio, implying reduction of FUNDC1 expression in EC cells inhibits the activation of autophagy. JC-1 was used to detect changes in mitochondrial membrane potential. In the normal state, JC-1 aggregates and forms polymers in the mitochondrial matrix, producing red fluorescence; once mitochondrial membrane potential decreases, JC-1 disaggregates to monomers, thereby producing green fluorescence^41^. In the present study, higher mitochondrial membrane potential levels were observed in FUNDC1-silenced HEC-1B cells, based on the JC-1 red/green ratio. These results indicate that inhibition of FUNDC1 ameliorates mitochondrial dysfunction in EC cells, suggesting a role of FUNDC1 in regulating mitophagy in EC.

Emerging evidence indicates that mitophagy not only contributes to tumorigenesis and cancer progression, but also plays a crucial part in cancer therapy resistance^42^. PINK1/Parkin-mediated mitophagy has been proposed to be the main mechanism of cisplatin resistance in ovarian cancer cells^43^. Chemoresistance to 5-fluorouracil in colorectal cancer and acquired sorafenib resistance in hepatocarcinoma cells is associated with aberrant methylation of BNIP3^44^, ^45^. Notably, depletion of FUNDC1 was found to enhance cell sensitivity to cisplatin and ionizing radiation in cervical cancer^23^. In the current study, EC patients with elevated expression of FUNDC1 were shown to have recurrence within one year following chemotherapy. In addition, knockdown of FUNDC1 enhances the cytotoxicity of carboplatin and paclitaxel in EC cells. These results uncover a distinctive role of FUNDC1 in chemoresistance in EC.

Previous studies suggest that ERK1/2 signal activation contributes to hydrogen peroxide-induced FUNDC1 upregulation in laryngeal cancer^22^. Our previous study illustrated that FUNDC1 promotes cell proliferation and migration through activating a calcium-NFATC1-BMI1 pathway in breast cancer^20^. Another study demonstrated that miR-137 could inhibit mitophagy by targeting FUNDC1 in breast cancer stem‑like cells^46^. Moreover, AFAP1L2-SRC-FUNDC1 pathway-dependent mitophagy has been demonstrated to be involved in mitigating sorafenib resistance in hepatocellular carcinoma cells^47^. Nevertheless, whether the above-mentioned molecular mechanisms contribute to FUNDC1-mediated mitophagy in EC progression and chemoresistance remains to be illustrated. Notably, NOD-like receptor signaling is the most significant signaling pathway for the FUNDC1-related genes in EC. This result was consistent with the finding that FUNDC1 is involved in NOD-like receptor X1 (NLRX1)-mediated mitophagy via a FUNDC1-NIPSNAP1/NIPSNAP2 pathway^48^, suggesting the potential role of NOD-like receptor signaling in FUNDC1-mediated mitophagy in EC.

Recent studies have revealed involvement of the tumor immune microenvironment in tumor pathology, EC included^49^, ^50^. For instance, memory CD4+ T cells, regulatory T cells, NK cells and dendritic cells have been suggested as prognostic tumor-infiltrating immune cells for EC patients^51^. Tumor-infiltrating MDSCs have been correlated with cancer stem cell induction and progression, chemo- and radio- resistance and even short survival in endometrial cancer^52^, ^53^. Similar to previous studies, immune infiltration analysis in our study indicates that FUNDC1 expression is associated with the infiltration of tumor-infiltrating lymphocytes, MDSCs, NK cells, pDC cells, and neutrophils in EC, suggesting the important roles of FUNDC1 in tumor immunology. Recent studies highlight the critical role of γδ T cells in killing cancer cells and predicting outcomes across a number of malignancies^54^, ^55^. However, γδ T cells in EC have not been well characterized. The current study shows that FUNDC1 is negatively associated with the infiltration of γδ T cells, which is consistent with the general consensus. Whether blocking FUNDC1 could enhance EC cell susceptibility to γδ T cell-mediated killing should be studied further.

However, there are some limitations in interpreting these research findings. Firstly, as a single-center retrospective study, the relatively small number of EC cases included is an inherent limitation that might lead to selection bias. Secondly, the effects of FUNDC1 on malignant phenotypes and cell viability in response to chemotherapeutic drugs were only verified in one EC cell model through knockdown of FUNDC1. Therefore, employing more EC cell lines and xenograft models to verify the role of FUNDC1 in EC progression and chemoresistance by upregulation of FUNDC1 should be carried out in subsequent research. Thirdly, a comprehensive understanding of the molecular mechanism underlying FUNDC1-mediated mitophagy in EC carcinogenesis remains to be elucidated.

## Conclusions

In conclusion, this study demonstrates the predictive value of FUNDC1 in prognosis and chemoresistance in EC patients. Moreover, our research highlights the role of FUNDC1-mediated mitophagy in malignant progression and chemotherapeutic resistance in EC cells. Therefore, FUNDC1 might serve as a candidate biomarker for prognostic prediction and as a promising target for chemoresistance intervention in EC.

## Supplementary Material

Supplementary table.

## Figures and Tables

**Table 1:** Correlation between FUNDC1 and HIF-1α expression and clinicopathological features of EC.

Characteristics	FUNDC1 expression		*Χ* ^2^	*P*-value	HIF-1α expression		*Χ* ^2^	*P*-value
	Low expression	High expression			Low expression	High expression		
**Age, n (%)**								
≤50	57 (19.8%)	38 (13.2%)	1.72	0.19	64 (22.2%)	31 (10.8%)	0.12	0.73
>50	100 (34.7%)	93 (32.3%)			126(43.7%)	67 (23.3%)		
Missing	0	0			0	0		
**BMI, n (%)**								
<28	106 (41.9%)	98 (38.7%)	0.02	0.89	136 (53.8%)	68 (26.9%)	0.03	0.86
≥28	26 (10.3%)	23 (9.1%)			32 (12.6%)	17 (6.7%)		
Missing	25	10			22	13		
**CA125, n (%)**								
<35	94 (38.5%)	74 (30.3%)	3.29	0.07	118 (48.4%)	50 (20.5%)	2.87	0.09
≥35	33 (13.5%)	43 (17.7%)			45 (18.4%)	31 (12.7%)		
Missing	30	14			27	17		
**LDH** ^a^ **, n (%)**								
<221.7	137 (48.2%)	114 (40.2%)	0.00	1.00	164 (57.8%)	87 (30.6%)	0.02	0.80
≥221.7	18 (6.3%)	15 (5.3%)			22 (7.7%)	11 (3.9%)		
Missing	2	2			4	0		
**Menopause,** **n (%)**								
No	74 (25.7%)	48 (16.7%)	3.22	0.07	84 (29.2%)	38 (13.2%)	0.78	0.38
Yes	83 (28.8%)	83 (28.8%)			106 (36.8%)	60 (20.8%)		
Missing	0	0			0	0		
**Hypertension,** **n (%)**								
No	101 (35.6%)	74 (26.0%)	1.81	0.18	114 (40.1%)	61 (21.5%)	0.41	0.52
Yes	54 (19.0%)	55 (19.4%)			75 (26.4%)	34 (12.0%)		
Missing	2	2			1	3		
**Diabetes, n (%)**								
No	135 (47.5%)	111 (39.1%)	0.07	0.80	165 (58.1%)	81 (28.5%)	0.23	0.63
Yes	20 (7.1%)	18 (6.3%)			24 (8.5%)	14 (4.9%)		
Missing	2	2			1	3		
**FIGO stage**^b^**,** **n (%)**								
Early	110 (38.2%)	91 (31.6%)	0.01	0.91	137 (47.6%)	64 (22.2%)	1.42	0.23
Advanced	47 (16.3%)	40 (13.9%)			53 (18.4%)	34 (11.8%)		
Missing	0	0			0	0		
**Tumor grade,** **n (%)**								
G1	36 (13.7%)	37 (14.2%)	1.13	0.29	49 (18.7%)	24 (9.2%)	0.23	0.88
G2+G3	107 (40.8%)	82 (31.3%)			125 (47.7%)	64 (24.4%)		
Missing	14	12			16	10		
**Lymph node invasion, n (%)**								
Negative	142 (49.3%)	112 (38.9%)	1.68	0.20	170 (59.0%)	84 (29.2%)	0.88	0.35
Positive	15 (5.2%)	19 (6.6%)			20 (6.9%)	14 (4.9%)		
Missing	0	0			0	0		
**Depth of myometrial invasion, n (%)**								
<1/2	133 (46.2%)	103 (35.8%)	1.79	0.18	160 (55.6%)	76 (26.4%)	1.94	0.16
≥1/2	24 (8.3%)	28 (9.7%)			30 (10.4%)	22 (7.6%)		
Missing	0	0			0	0		
**Cervical invasion, n (%)**								
No	136 (47.2%)	108 (37.5%)	0.97	0.33	168 (58.3%)	76 (26.4%)	**5.90**	**0.02**
Yes	21 (7.3%)	23 (8.0%)			22 (7.65%)	22 (7.65%)		
Missing	0	0			0	0		
**Adnexa invasion, n (%)**								
No	142 (49.3%)	118 (41.0%)	0.01	0.92	173 (60.1%)	87 (30.2%)	0.33	0.55
Yes	15 (5.2%)	13 (4.5%)			17 (5.9%)	11 (3.8%)		
Missing	0	0			0	0		
**Ascites Positive, n (%)**								
No	153 (53.1%)	128 (44.5%)	0.02	0.89	186 (64.6%)	95 (33.0%)	0.01	0.92
Yes	4 (1.4%)	3 (1.0%)			4 (1.4%)	3 (1.0%)		
Missing	0	0			0	0		
**ER expression** ^c^ **, n (%)**								
Low	36 (14.2%)	33 (13.0%)	0.54	0.46	44 (17.4%)	25 (9.8%)	0.17	0.69
High	106 (41.7%)	79 (31.1%)			123 (48.4%)	62 (24.4%)		
Missing	15	19			23	11		
**PR expression** ^d^ **, n (%)**								
Low	42 (16.5%)	38 (15.0%)	0.55	0.46	55 (21.7%)	25 (9.8%)	0.47	0.49
High	100 (39.4%)	74 (29.1%)			112 (44.1%)	62 (24.4%)		
Missing	15	19			23	11		

^a^ Lactate dehydrogenase. ^b^ Early stage: patients with stage I and II; advanced stage: patients with stage III and IV. ^c^ Estrogen receptor expression was set as low expression (-~+), and high expression (++~+++). ^d^ Progesterone receptor expression was set as low expression (-~+), and high expression (++~+++). Missing cases: Some of clinical data is missing.

**Table 2:** Univariate and multivariate analysis for overall survival.

Variables	Univariate analysis		Multivariate analysis	
	HR (95% CI)	*P*-value	HR (95% CI)	*P*-value
Age (≥50 vs <50)	1.93 (0.93-4.01)	0.079		
BMI^a^ (≥28 vs <28)	0.57 (0.22-1.44)	0.232		
CA125 (≥35 vs <35)	4.66 (2.36-9.20)	**<0.001**		
LDH^b^ (≥221.7 vs <221.7)	3.40 (1.75-6.61)	**<0.001**		
Menopause (yes vs no)	1.75 (0.92-3.35)	0.089		
Hypertension (yes vs no)	1.17 (0.64-2.16)	0.612		
Diabetes (yes vs no)	1.53 (0.71-3.31)	0.279		
FIGO stage^c^ (advanced vs early)	8.51 (4.30-16.85)	**<0.001**	4.51 (1.47-13.89)	**0.009**
Tumor grade (G2+G3 vs G1)	2.03 (0.85-4.85)	0.112		
Lymphatic invasion (positive vs negative)	6.54 (3.58-11.96)	**<0.001**	2.72 (1.09-6.81)	**0.032**
Depth of myometrial invasion (≥1/2 vs <1/2)	5.45 (3.02-9.85)	**<0.001**	3.11 (1.38-6.97)	**0.006**
Cervical invasion (yes vs no)	4.52 (2.48-8.24)	**<0.001**		
Adnexa invasion (yes vs no)	2.30 (1.07-4.94)	**0.033**		
Ascites (yes vs no)	12.55 (4.90-32.13)	**<0.001**	12.69 (3.36-47.99)	**<0.001**
ER expression^d^ (high vs low)	0.35 (0.18-0.69)	**0.002**		
PR expression^e^ (high vs low)	0.53 (0.27-1.04)	0.065		
FUNDC1 expression (high vs low)	2.27 (1.23-4.19)	**0.009**	2.46(1.13-5.35)	**0.023**
HIF-1α expression (high vs low)	1.85 (1.02-3.35)	**0.041**		
Postoperative complications (yes vs no)	1.31 (0.69-2.51)	0.408		

^a^ Body mass index. ^b^ Lactate dehydrogenase. ^c^ Early stage: patients with stage I and II; advanced stage: patients with stage III and IV. ^d^ Estrogen receptor expression was set as low expression (-~+), and high expression (++~+++). ^e^ Progesterone receptor expression was set as low expression (-~+), and high expression (++~+++).

**Table 3:** Univariate and multivariate analysis for progression-free survival.

Variables	Univariate analysis		Multivariate analysis			
	HR (95% CI)	*P*-value	HR (95% CI)		*P*-value	
Age (≥50 vs <50)	1.92(0.99-3.74)	0.054				
BMI^a^ (≥28 vs <28)	0.66 (0.30-1.47)	0.312				
CA125 (≥35 vs <35)	4.17 (2.27-7.66)	**<0.001**				
LDH^b^ (≥221.7 vs <221.7)	2.80 (1.47-5.34)	**0.002**				
Menopause (yes vs no)	1.56 (0.88-2.78)	0.131				
Hypertension (yes vs no)	0.91 (0.52-1.61)	0.745				
Diabetes (yes vs no)	1.63 (0.82-3.26)	0.166				
FIGO stage^c^ (advanced vs early)	7.27 (3.99-13.23)	**<0.001**	5.36 (2.41-11.92)			**<0.001**
Tumor grade (G2+G3 vs G1)	2.16 (0.97-4.82)	0.061				
Lymphatic invasion (positive vs negative)	5.07 (2.89-8.89)	**<0.001**				
Depth of myometrial invasion (≥1/2 vs <1/2)	4.58 (2.66-7.87)	**<0.001**	3.08 (1.49-6.37)			**0.002**
Cervical invasion (yes vs no)	4.37 (2.52-7.59)	**<0.001**				
Adnexa invasion (yes vs no)	2.50 (1.256-4.98)	**0.009**				
Ascites (yes vs no)	9.64 (3.80-24.42)	**<0.001**	7.53 (2.17-26.12)			**0.001**
ER expression^d^ (high vs low)	0.49 (0.28-0.87)	**0.014**				
PR expression^e^ (high vs low)	0.59 (0.33-1.03)	0.065				
FUNDC1 expression (high vs low)	2.21 (1.27-3.86)	**0.005**	2.67 (1.32-5.39)			**0.006**
HIF-1α expression (high vs low)	1.28 (0.74-2.22)	0.384				
Postoperative complications (yes vs no)	1.26 (0.70-2.26)	0.443				
						

^a^ Body mass index. ^b^ Lactate dehydrogenase. ^c^ Early stage: patients with stage I and II; advanced stage: patients with stage III and IV. ^d^ Estrogen receptor expression was set as low expression (-~+), and high expression (++~+++). ^e^ Progesterone receptor expression was set as low expression (-~+), and high expression (++~+++).

## Abbreviations

EC: endometrial carcinoma
OS: overall survival
PFS: progression-free survival
TCGA: The Cancer Genome Atlas
FUNDC1: FUN14 domain-containing protein 1
HIF-1α: hypoxia inducible factor-1α
ROS: reactive oxygen species
BNIP3: BCL2 and adenovirus E1B 19-kDa-interacting protein 3
LC3: microtubule-associated protein light chain 3
FIGO: Federation of International of Gynecology and Obstetrics
PBS: phosphate-buffered saline
FBS: fetal bovine serum
shRNA: Short hairpin RNA
CCK8: Cell Counting Kit-8
HR: hazard ratios
BMI: body mass index
LDH: lactate dehydrogenase
ER: estrogen receptor
PR: progesterone receptor
CBP: carboplatin
PTX: paclitaxel
NK cells: natural killer cells
Tem cells: effector memory T cells
pDC cells: pre-dendritic cells
MDSCs: myeloid-derived suppressor cells
