# Supplementary material for: FUNDC1 predicts Poor Prognosis and promotes Progression and Chemoresistance in Endometrial Carcinoma
Source: J Cancer. 2024 Oct 21;15(20):6490–504. doi: 10.7150/jca.96877 (PMC11632979; doi:10.7150/jca.96877)
Supplement: Supplementary file 1 — Supplementary table. [file jcav15p6490s1.pdf]

Supplementary Tables

Table S1. Correlation between FUNDC1 and HIF-1α expression and clinicopathological features of EC in TCGA.

Characteristics	FUNDC1 expression		χ^2	*P*-value	HIF-1α expression		χ^2	*P*-value
	Low expression	High expression			Low expression	High expression		
**Age, n (%)**								
≤50	33 (8.3%)	11 (2.8%)	**5.38**	**0.02**	6 (1.5%)	38 (9.5%)	1.25	0.26
>50	202 (50.5%)	154 (38.4%)			74 (18.5%)	282 (70.5%)		
Missing	0	3			0	3		
**FIGO stage[a], n (%)**								
Early	185 (46.0%)	136 (33.7%)	0.30	0.58	64 (15.9%)	257 (63.8%)	0.01	0.93
Advanced	50 (12.4%)	32(7.9%)			16(3.9%)	66(16.4%)		
Missing	0	0			0	0		
**Tumor grade, n (%)**								
G1	61 (15.1%)	36 (8.9%)	1.10	0.29	20 (5.0%)	77 (19.1%)	0.05	0.83
G2+G3	174 (43.2%)	132 (32.8%)			60 (14.9%)	246 (61.0%)		
Missing	0	0			0	0		
**Lymph node invasion, n (%)**								
Negative	203 (50.4%)	152 (37.7%)	1.56	0.21	72 (17.9%)	283 (70.2%)	0.38	0.56
Positive	32 (7.9%)	16 (4.0%)			8 (2.0%)	40 (9.9%)		
Missing	0	0			0	0		

[a] Early stage: patients with stage I and II; advanced stage: patients with stage III and IV. Missing cases: Some of TCGA clinical data are missing.
